# Guideline-concordant treatment and clinical outcomes in older patients with endometrial cancer in Japan: a retrospective single-institution study

**DOI:** 10.1007/s10147-026-03113-z

**Published:** 2026-07-23

**Authors:** Kanako Kasuya, Naoko Komura, Satoshi Nakagawa, Tsuyoshi Takiuchi, Eiji Kobayashi, Kae Hashimoto, Yutaka Ueda, Kenjiro Sawada, Michiko Kodama

**Affiliations:** 1 Department of Obstetrics and Gynecology, Osaka University Graduate School of Medicine, 2-2 Yamadaoka, Suita, Osaka 565-0871 Japan; 2 Department of Obstetrics and Gynecology, Faculty of Medicine, Oita University, Oita, Japan; 3 Department of Advanced Prevention and Health Sciences, Wakayama Medical University, Wakayama, Japan; 4 Department of Obstetrics and Gynecology, Ehime University Graduate School of Medicine, Ehime, Japan

**Keywords:** Endometrial cancer, Older patients, Guideline-concordant treatment, Surgical staging, Adjuvant chemotherapy, Progression-free survival

## Abstract

**Background:**

To evaluate achievement of Japanese guideline-concordant treatment (J-GCT) and examine its association with clinical outcomes among older patients with endometrial cancer.

**Methods:**

We retrospectively reviewed 130 patients aged ≥ 70 years diagnosed between 2010 and 2019. J-GCT was defined as receipt of primary surgery, standard surgical staging including lymph node assessment when indicated, and risk-adapted adjuvant chemotherapy. Progression-free survival (PFS) and overall survival (OS) were analyzed using Kaplan–Meier methods and log-rank tests. Multivariable Cox models evaluated factors associated with PFS.

**Results:**

J-GCT achievement was significantly lower in patients aged ≥ 75 years than in those aged 70–74 years (28.1% vs. 71.2%, *P* < 0.001). Patients aged ≥ 75 years less frequently underwent primary surgery (84.2% vs 97.3%, *P* = 0.011) and lymph node assessment (45.8% vs 80.3%, *P* < 0.001). Adjuvant chemotherapy for intermediate- or high-risk disease was less frequently administered in patients aged ≥ 75 years (38.7% vs 86.4%, *P* < 0.001). Perioperative complications were infrequent, and no treatment-related deaths were observed. J-GCT achievement was associated with improved PFS (*P* < 0.001) and OS (*P* = 0.004). In multivariable analysis adjusting for age and selected clinicopathologic factors, failure to achieve J-GCT remained associated with inferior PFS (adjusted hazard ratio 3.20, 95% confidence interval 1.43–7.15; *P* = 0.005).

**Conclusion:**

J-GCT achievement was lower among patients aged ≥ 75 years, particularly with respect to lymph node assessment and risk-adapted adjuvant chemotherapy. Failure to achieve J-GCT was associated with inferior PFS after adjustment, although this group included heterogeneous treatment patterns with potentially different prognostic implications. These findings support treatment decision-making based on structured clinical evaluation rather than chronological age alone.

**Supplementary information:**

The online version contains supplementary material available at 10.1007/s10147-026-03113-z.

## Introduction

With rapid population aging in Japan, the number of older patients with endometrial cancer encountered in clinical practice is increasing [1]. In this population, treatment decisions are often individualized based on comorbidities, perioperative risk, and treatment tolerability. Accordingly, clarifying how closely management conforms to guideline recommendations and how such concordance relates to prognosis among older patients is clinically important.

Primary surgery is the standard treatment for endometrial cancer. Guideline-recommended care includes appropriate surgical staging with lymph node assessment when indicated and risk-adapted adjuvant therapy based on stage, histology, and other clinicopathologic risk factors [2]. Guideline-concordant treatment (GCT) indicates whether patients received treatment components recommended by clinical practice guidelines according to their disease stage, histology, and risk profile. Previous studies have used GCT as a quality-of-care metric to assess adherence to recommended endometrial cancer treatment and its association with survival outcomes in routine clinical practice [3]. Population-based studies have shown lower concordance with increasing age and suggested that outcomes in older patients reflect tumor characteristics, patient-related factors, and treatment selection [4, 5]. However, data from Japan evaluating GCT as a patient-level composite measure in endometrial cancer remain limited.

In this study, we defined Japanese guideline-concordant treatment (J-GCT) and examined its association with outcomes among patients aged 70 years and older with endometrial cancer, focusing on primary surgery, standard surgical management with lymph node assessment when indicated, and risk-adapted adjuvant chemotherapy for intermediate- and high-risk disease.

## Patients and methods

### Study design and population

This retrospective observational study was conducted at Osaka University Hospital. We reviewed patients aged 70 years and older with histologically confirmed endometrial cancer diagnosed at our institution between January 2010 and October 2019. Patients were excluded if they had uterine carcinosarcoma, uterine sarcoma, other non-endometrial uterine malignancies, or missing clinical outcome data. Patients were categorized into two age groups (70–74 and ≥ 75 years) for comparison. The study was approved by the institutional review board of Osaka University Hospital (IRB No. 22408).

### Clinicopathologic variables and risk classification

Baseline variables included age, body mass index, parity, comorbidities, Eastern Cooperative Oncology Group performance status (ECOG PS), and activities of daily living (ADL). ADL was assessed at admission using a nurse-performed discharge planning assessment. Any ADL assistance was defined as requiring help in ≥ 1 of the following domains: feeding, dressing, transferring, toileting, and bathing/showering; continence was not assessed. Ambulation aid use was defined as use of a cane or wheelchair at admission. Tumor characteristics included the International Federation of Gynecology and Obstetrics (FIGO) 2009 stage and histologic subtype/grade. Treatment-related variables included initial treatment modality (surgery or non-surgical management), surgical approach, performance of lymph node assessment (pelvic and/or paraaortic lymphadenectomy), and perioperative complications. Perioperative complications were defined as Clavien–Dindo grade II or higher events occurring intraoperatively or within 30 days after surgery and were abstracted from medical records.

Recurrence risk classification (low, intermediate, high) was assigned according to the Japan Society of Gynecologic Oncology (JSGO) 2018 guidelines for treatment of uterine body neoplasms [6], using FIGO 2009 stage and clinicopathologic factors, including depth of myometrial invasion, lymphovascular space invasion, histologic subtype/grade, cervical stromal invasion, and extrauterine disease, and was evaluated among patients who underwent primary surgery.

### Adjuvant therapy

Adjuvant chemotherapy was generally administered to patients classified as intermediate or high risk according to the Japanese guideline criteria. Chemotherapy regimens included TEC (paclitaxel, epirubicin, and carboplatin), paclitaxel plus carboplatin administered every 3 weeks or weekly, and other regimens. The regimen, schedule, and number of cycles were selected and modified as needed at the treating physician’s discretion, based on the patient’s general condition, comorbidities, and treatment tolerability. Chemotherapy completion, discontinuation, dose reduction, treatment delays (≥ 4 weeks), regimen modifications, and adverse effects were abstracted from medical records. Adverse events were assessed using the Common Terminology Criteria for Adverse Events (CTCAE), version 4.03.

### Definition of Japanese guideline-concordant treatment (J-GCT)

We defined J-GCT achievement as meeting all of the following three components: [1] receipt of primary surgery as the initial treatment, [2] achievement of standard surgical management, and [3] administration of adjuvant chemotherapy for patients classified as intermediate- or high-risk. This adjuvant component was defined as chemotherapy because postoperative adjuvant management in Japan primarily consists of chemotherapy rather than radiotherapy. Standard surgical management (hereafter, standard surgery) was defined as completion of guideline-recommended surgical staging at our institution, including lymph node assessment when indicated. Indications for pelvic and paraaortic lymphadenectomy were determined based on preoperative imaging and endometrial biopsy findings, and, as needed, intraoperative frozen-section assessment of myometrial invasion and cervical stromal involvement. Specifically, pelvic lymphadenectomy was omitted for endometrioid grade 1 tumors with no myometrial invasion, whereas it was performed for any myometrial invasion (including < 50% invasion), grade 2–3, or non-endometrioid high-risk histology. Paraaortic lymphadenectomy was added for deep myometrial invasion (≥ 50%), grade 3, non-endometrioid high-risk histology, or cervical stromal involvement. Failure to achieve J-GCT was defined as omission of any component, including non-surgical initial management, non-standard surgery, or omission of adjuvant chemotherapy in intermediate- or high-risk disease.

### Outcome and follow-up

The primary outcome was progression-free survival (PFS), defined as the time from initiation of initial management to the first documented clinical and/or radiographic disease progression or death from any cause. Patients without an event were censored at the date of last follow-up. The secondary outcome was overall survival (OS), defined as the time from initiation of initial management to death from any cause. Patients who were alive at last follow-up were censored on that date.

For patients managed with best supportive care, initiation was defined as the date when best supportive care was selected as the initial treatment plan. Follow-up information was obtained from medical records.

### Statistical analysis

Categorical variables were compared using the chi-square test or Fisher’s exact test, and continuous variables using the Wilcoxon rank-sum test, as appropriate. PFS and OS were estimated using the Kaplan–Meier method and compared using the log-rank test. Cox proportional hazards models were used to evaluate factors associated with PFS. Variables in the multivariable models were selected based on clinical relevance and prior literature, rather than univariate statistical significance alone. *P* values < 0.05 were considered statistically significant. All statistical analyses were performed using JMP® Pro version 15 (SAS Institute Inc., NC, USA) and GraphPad Prism version 10 (GraphPad Software, CA, USA).

## Results

### Patient characteristics

A total of 130 patients aged 70 years and older with histologically confirmed endometrial cancer were included, with 73 patients aged 70–74 years and 57 patients aged ≥ 75 years (Table 1). Median age was 72 years (range, 70–74) in the 70–74 group and 79 years (range, 75–92) in the ≥ 75 group. Body mass index was similar between groups (*P* = 0.437). The proportion with ECOG PS 0 was lower in the ≥ 75 group than in the 70–74 group (68.4% vs 87.7%; *P* = 0.020). Functional status measures also differed by age: ambulation aid use was more common in the ≥ 75 group than in the 70–74 group (15.8% vs 2.7%; *P* = 0.011), whereas any ADL assistance was uncommon and did not significantly differ between groups (10.5% vs 2.7%; *P* = 0.137). The proportion of patients with any recorded comorbidity did not significantly differ between groups (35.1% vs 24.7%; *P* = 0.244). FIGO stage distribution did not differ between age groups (*P* = 0.151), and stage I disease was the most common stage in both groups (63.2% vs 71.2%). Endometrioid carcinoma grade 1/2 was the most common histology in both groups (54.4% vs 61.6%).

**Table 1:** Clinicopathological characteristics of patients by age group

		Age ≥ 75 (N = 57) N (%)	Age 70–74 (N = 73) N (%)	*P*-value
Age (years)	Median (Range)	79 (75–92)	72 (70–74)	
BMI (kg/m^2^)	Mean ± SD	22.8 ± 4.5	23.5 ± 3.6	0.437
ECOG PS	0	39 (68.4)	64 (87.7)	0.020
	1	15 (26.3)	8 (11.0)	
	≥ 2	3 (5.3)	1 (1.4)	
ADL	Any ADL assistance^a^	6 (10.5)	2 (2.7)	0.137
	Ambulation aid use^b^	9 (15.8)	2 (2.7)	0.011
Parity	Nulliparous	11 (19.3)	6 (8.2)	0.072
Comorbidities	Any	20 (35.1)	18 (24.7)	0.244
(Detailed categories)	Hypertension	10 (17.5)	10 (13.7)	
	Diabetes	5 (8.8)	6 (8.2)	
	Lipid disorders	6 (10.5)	8 (11.0)	
	Cognitive impairment	3 (5.3)	2 (2.7)	
	Congestive heart failure	3 (5.3)	1 (1.4)	
	Chronic renal failure	1 (1.8)	1 (1.4)	
FIGO stage^c^	I	36 (63.2)	52 (71.2)	0.151
	II	9 (15.8)	3 (4.1)	
	III	5 (8.8)	9 (12.3)	
	IV	6 (10.5)	9 (12.3)	
Histology	High-risk histology^d^	12 (21.1)	21 (28.8)	0.417
(Detailed categories)	Endometrioid carcinoma^e^			
	Grade 1/2	31 (54.4)	45 (61.6)	
	Grade 3	4 (7.0)	6 (8.2)	
	Serous carcinoma	6 (10.5)	12 (16.4)	
	Clear cell carcinoma	3 (5.3)	4 (5.5)	
	Mucinous carcinoma	1 (1.8)	0	
	Mixed carcinoma	3 (5.3)	5 (6.8)	
	Unknown	4 (7.0)	1 (1.4)	

BMI, body mass index; ECOG PS, Eastern Cooperative Oncology Group performance status; ADL, activities of daily living; FIGO, International Federation of Gynecology and Obstetrics

^a^Any ADL assistance was defined as requiring help in ≥ 1 ADL domain at admission

^b^Ambulation aid use was defined as cane/wheelchair use at admission

^c^FIGO stage was not documented for 1 patient

^d^High-risk histology was defined as serous, clear cell, or mixed carcinoma with any serous or clear cell component

^e^Endometrioid grade was not specified in 5 patients aged ≥ 75 years

### Japanese guideline–concordant treatment (J-GCT) achievement, surgical feasibility, and adjuvant therapy gaps by age

Overall, J-GCT achievement was lower in patients aged ≥ 75 years than in those aged 70–74 years (28.1% vs 71.2%; *P* < 0.001). Most patients underwent primary surgery as the initial treatment, although the proportion was lower in those aged ≥ 75 years than in those aged 70–74 years (84.2% vs 97.3%; *P* = 0.011). Among patients undergoing surgery, surgical approach did not differ significantly between age groups (laparotomy: 64.6% vs 63.4%; laparoscopy: 35.4% vs 33.8%; *P* = 0.723). The overall rate of perioperative complications was low, with no statistically significant difference between groups (4.2% vs 7.0%; *P* = 0.700), and thromboembolic complications were the most frequent (deep vein thrombosis: 2.1% vs 2.7%; pulmonary embolism: 0% vs 4.1%). There were no cases of failure to discharge home in either age group (Table 2).

**Table 2:** Primary treatment and perioperative outcomes by age group

		Age ≥ 75 (N = 57) N (%)	Age 70–74 (N = 73) N (%)	*P*-value
J-GCT	Achieved	16 (28.1)	52 (71.2)	< 0.001
	Not achieved	41 (71.9)	21 (28.8)	
Primary treatment				
Surgery		48 (84.2)	71 (97.3)	0.011
Non-surgery	Chemotherapy alone	3 (5.2)	1 (1.3)	
	Radiotherapy alone	1 (1.8)	0	
	Best supportive care	5 (8.8)	1 (1.3)	
Details of surgery^a^				
Type of surgery	Laparotomy	31 (64.6)	45 (63.4)	0.723
	Laparoscopy	17 (35.4)	24 (33.8)	
	Robotic-assisted	0	2 (2.8)	
Any lymphadenectomy (PEN and/or PAN)		22 (45.8)	57 (80.3)	< 0.001
	PEN	22 (45.8)	57 (80.3)	
	PAN	6 (12.5)	26 (36.6)	
Completion of standard surgery^b^		18 (37.5)	56 (78.9)	< 0.001
Perioperative complications^c^				
	Any	2 (4.2)	5 (7.0)	0.700
(Detailed categories)	Intraoperative blood transfusion	0	1 (1.4)	
	Deep vein thrombosis	1 (2.1)	2 (2.7)	
	Pulmonary embolism	0	3 (4.1)	
	Ileus	1 (2.1)	2 (2.7)	
Failure to discharge home^d^		0	0	NC

J-GCT, Japanese guideline-concordant treatment; PEN, pelvic lymphadenectomy; PAN, paraaortic lymphadenectomy; NC, not calculated (no events in either group)

^a^Percentages are calculated among patients who underwent surgery (Age ≥ 75, n = 48; Age 70–74, n = 71)

^b^Standard surgery was defined as guideline-recommended surgical staging, including lymph node assessment when indicated

^C^Any complication is counted per patient; multiple events in the same patient were counted once

^d^Failure to discharge home was defined as discharge to a facility or transfer to another hospital, including in-hospital death

Component-level evaluation demonstrated age-associated gaps in guideline-recommended treatment delivery across multiple steps (Tables 2, 3, and Fig. S1). First, non-surgical initial management was more common in the ≥ 75 group (15.8% vs 2.7%), including chemotherapy alone (5.2% vs 1.3%), radiotherapy alone (1.8% vs 0%), and best supportive care (8.8% vs 1.3%). Second, among surgical patients, lymph node assessment (pelvic and/or paraaortic lymphadenectomy) was performed less often in the ≥ 75 group (45.8% vs 80.3%; *P* < 0.001), with paraaortic assessment particularly less frequent (12.5% vs 36.6%). Accordingly, the proportion undergoing standard surgery was lower in the ≥ 75 group (37.5% vs 78.9%) (Table 2). J-GCT achievement stratified by recurrence risk group is shown in Table S1.

**Table 3:** Recurrence risk classification and adjuvant chemotherapy among patients who underwent surgery

		Age ≥ 75 (N = 48) N (%)	Age 70–74 (N = 71) N (%)	*P*-value
Recurrence risk classification	Low	17 (35.4)	27 (38.0)	0.748
	Intermediate	14 (29.2)	16 (22.5)	
	High	17 (35.4)	28 (39.4)	
Administration of adjuvant chemotherapy^a^		12 / 31 (38.7)	38 / 44 (86.4)	< 0.001
Adjuvant chemotherapy^b^	Intermediate	3 / 14 (21.4)	12 / 16 (75.0)	
	High	9 / 17 (52.9)	26 / 28 (92.9)	
No adjuvant chemotherapy^c^	Due to patient preference	3 (15.8)	3 (50.0)	
	Due to advanced age	7 (36.8)	1 (16.7)	
	Due to PS	1 (5.3)	0 (0)	
	Due to comorbidities	1 (5.3)	1 (16.7)	
	Unknown	7 (36.8)	1 (16.7)	
Adjuvant chemotherapy regimen^d^	TEC	3 (25.0)	17 (44.7)	
	TC triweekly	8 (66.7)	11 (28.9)	
	TC weekly	1 (8.3)	5 (13.2)	
	Others^e^	0	5 (13.2)	
Completion of systemic therapy^d^	Completion	5 (41.7)	23 (60.5)	0.324
	Treatment discontinuation	4 (33.3)	11 (28.9)	
	Dose reduction	2 (16.7)	4 (10.5)	
	Treatment delay (≥ 4 weeks)	1 (8.3)	1 (2.6)	
	Regimen modification	1 (8.3)	2 (5.3)	
Advers events^d, f^	Any	7 (58.3)	32 (84.2)	0.105
(Detailed categories)	Neutropenia	7 (58.3)	28 (73.7)	
	Thrombocytopenia	0	1 (2.6)	
	Anemia	0	8 (21.1)	
	Febrile neutropenia	0	3 (7.9)	
	Nausea	1 (8.3)	0	
	Hypokalemia	0	1 (2.6)	

This table includes only patients who underwent primary surgery

PS, performance status; TEC, paclitaxel, epirubicin, and carboplatin; TC, paclitaxel and carboplatin

^a^Percentages are calculated among patients with intermediate- and high-risk classification

^b^Percentages are calculated within each risk category (Intermediate or High)

^c^Percentages are calculated among patients who did not receive adjuvant chemotherapy

^d^Percentages are calculated among patients who received adjuvant chemotherapy

^e^Others: Combination of paclitaxel, doxorubicin, and carboplatin, combination of docetaxel and carboplatin, and combination of doxorubicin and cisplatin

^f^Adverse events of Common Terminology Criteria for Adverse Events grade ≥ 3 are shown

Among patients who underwent primary surgery, recurrence risk distribution (low, intermediate, high) did not differ by age (*P* = 0.748) (Table 3). However, the proportion receiving adjuvant chemotherapy in intermediate- and high-risk disease was markedly lower in the ≥ 75 group (38.7% vs 86.4%; *P* < 0.001), with similar gaps observed in intermediate-risk (21.4% vs 75.0%) and high-risk disease (52.9% vs 92.9%). Among patients who received adjuvant chemotherapy, treatment completion did not differ significantly between age groups (41.7% vs 60.5%; *P* = 0.324), and grade ≥ 3 adverse effects were documented in 58.3% vs 84.2% (*P* = 0.105), with neutropenia being the most common adverse event. No treatment-related deaths (CTCAE grade 5 adverse events) were observed. To further characterize non-achievement of J-GCT, we examined treatment patterns among the 62 patients who failed to achieve J-GCT. These patterns were heterogeneous and included non-surgical management in 11 patients (17.7%), standard surgery with indicated adjuvant chemotherapy omitted in 6 (9.7%), non-standard surgery with adjuvant chemotherapy administered in 13 (21.0%), non-standard surgery with indicated adjuvant chemotherapy omitted in 19 (30.6%), and non-standard surgery without an indication for adjuvant chemotherapy in 13 (21.0%). Reasons for non-completion of standard surgery are also summarized in Table S2.

### Prognostic impact of J-GCT and supportive analyses

**Fig. 1:**
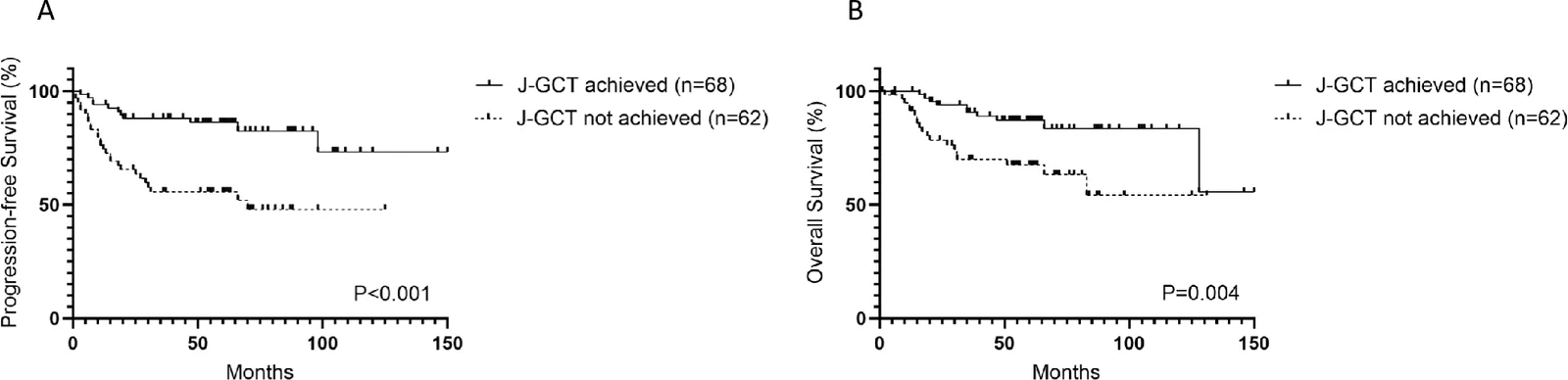
Kaplan–Meier survival curves by Japanese guideline–concordant treatment (J-GCT) in patients aged ≥ 70 years with endometrial cancer. (**A**) Progression-free survival. (**B**) Overall survival. *P* values were calculated using the log-rank test. Patients managed with best supportive care were included in the non-J-GCT group.

We next examined whether these age-associated differences in the delivery of guideline-recommended treatment were associated with differences in survival outcomes. To evaluate whether J-GCT stratified prognosis in this cohort, PFS was compared according to J-GCT achievement. Patients who achieved J-GCT had more favorable PFS than those who did not achieve J-GCT (*P* < 0.001) (Fig. 1A). OS was also more favorable among patients who achieved J-GCT (*P* = 0.004) (Fig. 1B). These analyses were based on overall J-GCT status, rather than on individual treatment components.

In exploratory and supportive analyses, we also examined survival by recurrence-risk categories within each age group. In patients aged 70–74 years, PFS and OS differed across risk categories (*P* = 0.009 and *P* = 0.021, respectively), whereas no significant differences were observed in patients aged ≥ 75 years (*P* = 0.851 and *P* = 0.146) (Fig. S2). PFS and OS were also significantly worse in the non-surgical group than in the surgical group (*P* < 0.001 for both) (Fig. S3). Among patients who underwent primary surgery and were classified as intermediate- or high-risk, PFS did not differ significantly by J-GCT achievement in patients aged ≥ 75 years (*P* = 0.161) or 70–74 years (*P* = 0.411), and OS also did not differ (*P* = 0.164 and *P* = 0.633, respectively) (Fig. S4).

### Multivariable analysis of factors associated with progression-free survival

To assess whether the association between J-GCT and PFS persisted after adjustment for selected clinicopathologic prognostic factors, multivariable Cox proportional hazards analyses were performed. We first adjusted for clinical factors (age group, ECOG performance status, FIGO stage, and histology) and then additionally adjusted for J-GCT. In the fully adjusted model including J-GCT, the association between age ≥ 75 years and PFS was attenuated (*P* = 0.136), whereas failure to achieve J-GCT remained associated with inferior PFS (adjusted hazard ratio [aHR] 3.20, 95% confidence interval [CI] 1.43–7.15; *P* = 0.005) (Table 4). In a sensitivity analysis excluding stage from the multivariable model, failure to achieve J-GCT remained significantly associated with inferior PFS (aHR 2.67, 95% CI 1.18–6.05; *P* = 0.018) (Table S3). Similar results were observed after excluding patients managed with best supportive care (aHR 2.83, 95% CI 1.24–6.44; *P* = 0.013) (Table S4).

**Table 4:** Risk factors associated with progression-free survival in all patients

	Univariate analysis		Multivariate analysis (clinical factors)		Multivariate analysis (clinical factors + J-GCT)	
Variables	HR (95% CI)	*P*-value	aHR (95% CI)	*P*-value	aHR (95% CI)	*P*-value
Age ≥ 75 years	2.22 (1.16–4.25)	0.016	2.82 (1.32–6.04)	0.007	1.87 (0.82–4.27)	0.136
ECOG PS (≥ 1 vs 0)	2.11 (1.07–4.20)	0.032	1.14 (0.50–2.60)	0.751	0.97 (0.41–2.29)	0.937
Advanced stage (III/IV)	5.60 (2.95–10.66)	< 0.001	4.90 (2.27–10.58)	< 0.001	5.37 (2.39–12.06)	< 0.001
High-risk histology^a^	3.84 (1.95–7.58)	< 0.001	2.57 (1.23–5.36)	0.012	2.29 (1.05–4.98)	0.036
Failure to achieve J-GCT	3.67 (1.81–7.44)	< 0.001			3.20 (1.43–7.15)	0.005

aHR, adjusted hazard ratio; CI, confidence interval; ECOG PS, Eastern Cooperative Oncology Group performance status; J-GCT, Japanese guideline-concordant treatment

Covariate data were missing for stage (n = 1) and high-risk histology (n = 5); patients with missing covariates were excluded from univariate and multivariable Cox models

^a^High-risk histology was defined as serous, clear cell, or mixed carcinoma with any serous or clear cell component

In supportive analyses among intermediate- or high-risk patients after primary surgery, PFS curves were numerically less favorable among patients who did not receive adjuvant chemotherapy, although the difference was not statistically significant (adjuvant, n = 50; no adjuvant, n = 25; *P* = 0.171) (Fig. S5). OS curves were similar between groups (*P* = 0.932) (Fig. S5). In an adjusted model, the association between omission of adjuvant chemotherapy and PFS was not statistically conclusive in this sample, although the HR was directionally unfavorable (aHR 2.37, 95% CI 0.78–7.14; *P* = 0.126) (Table S5).

## Discussion

### Summary of key findings

In this study, J-GCT achievement was substantially lower among patients aged ≥ 75 years than among those aged 70–74 years, driven mainly by gaps in lymph node assessment and risk-adapted adjuvant chemotherapy. Primary surgery was feasible with low perioperative complication rates, and no treatment-related deaths were observed. Failure to achieve J-GCT was associated with inferior PFS after adjustment for selected clinical and tumor factors, although this group included heterogeneous treatment patterns.

### Age-related declines in J-GCT and gaps in guideline-recommended treatment delivery

Consistent with prior Japanese data [7], we observed substantially lower J-GCT achievement among patients aged ≥ 75 years. Although the prevalence of recorded comorbidity was similar between age groups, functional measures differed, with poorer ECOG performance status and greater use of ambulation aids in the ≥ 75 group (Table 1). These findings suggest that de-intensification of surgical staging and/or adjuvant therapy may have been influenced more by functional age and perceived frailty than by documented comorbidities alone. Broader geriatric domains, including comorbidity burden, cognition, and social support, were not fully captured and may also have influenced treatment decisions [8].

### Feasibility and implications of surgery and lymph node assessment

Most patients underwent primary surgery with low perioperative complication rates, without significant differences by age, suggesting perioperative feasibility among selected older patients and consistent with prior reports [9]. However, lymph node assessment, particularly paraaortic assessment, was performed less frequently in patients aged ≥ 75 years, consistent with prior reports of less comprehensive staging with increasing age [10]. Less invasive approaches such as minimally invasive surgery and sentinel lymph node mapping may help preserve staging information while reducing surgical burden in selected older adults [11–16].

### Adjuvant chemotherapy gap in intermediate- or high-risk disease

A substantial gap in the delivery of risk-adapted adjuvant chemotherapy was observed among intermediate- or high-risk patients aged ≥ 75 years. Concerns about toxicity and competing mortality risks may contribute to omission in clinical practice. Notably, among patients who received adjuvant chemotherapy, completion rates and documented adverse events did not significantly differ by age group in our cohort (Table 3), suggesting that adjuvant chemotherapy may be feasible in carefully selected older patients. However, this may reflect treatment selection, highlighting the need for more standardized pretreatment evaluation and supportive care. Standardized pretreatment evaluation, including geriatric assessment tools such as G8 or VES-13, may help guide selection for adjuvant therapy and treatment intensity in older adults [17–20].

### J-GCT and prognostic stratification in older patients

In exploratory age-stratified analyses, postoperative recurrence-risk categories appeared to separate PFS more clearly in patients aged 70–74 years than in those aged ≥ 75 years (Fig. S2). This may reflect less comprehensive surgical staging and lower receipt of risk-adapted adjuvant chemotherapy, which could contribute to recurrence-risk misclassification and reduce the prognostic discrimination of postoperative clinicopathologic risk categories. In patients aged ≥ 75 years, relatively favorable OS despite PFS events in intermediate-risk disease may reflect treatable or indolent recurrences and the potential impact of post-recurrence therapy. These relative contributions could not be distinguished in this retrospective cohort.

The observed age-related differences in J-GCT achievement are consistent with prior data showing that older patients are less likely to receive guideline-indicated therapy and should not be excluded from diagnostic assessment or adjuvant treatment based on age alone [21, 22]. Current geriatric oncology guidance likewise supports incorporating life expectancy and noncancer mortality risk into treatment decisions [19].

Previous studies of treatment patterns and outcomes in older patients with endometrial cancer are summarized in Table S6. These studies generally support the importance of appropriate surgery, staging, and adjuvant therapy in older patients. However, direct comparisons of GCT adherence rates across studies require caution because guideline definitions and treatment practices differ across settings, particularly regarding the role of risk-adapted adjuvant chemotherapy in Japan versus adjuvant radiotherapy in many international studies.

In multivariable analyses, the association between age and PFS was attenuated after adjustment for J-GCT, whereas failure to achieve J-GCT remained associated with inferior PFS after adjustment for selected clinical and tumor factors (Table 4). As postoperative stage may partly depend on lymph node assessment included in the J-GCT definition, we performed a sensitivity analysis excluding stage from the multivariable model, which showed similar findings (Table S3). The association also remained significant after exclusion of patients managed with best supportive care (Table S4).

Nevertheless, failure to achieve J-GCT should be interpreted as a composite measure. Patients who failed to achieve J-GCT included clinically heterogeneous treatment patterns, and poorer outcomes in this group may not be attributable equally to all components. These outcomes may have been driven at least in part by non-surgical initial management, whereas the independent contribution of other components, including omission of adjuvant chemotherapy, remained uncertain because of the limited sample size.

In supportive analyses among intermediate- or high-risk patients, omission of adjuvant chemotherapy showed a numerically unfavorable pattern for PFS, but the association was not statistically conclusive in this sample (Fig. S5 and Table S5). OS curves were similar between groups. Among patients who omitted adjuvant chemotherapy and subsequently recurred (n = 8), post-recurrence treatment was administered to 4 patients (50.0%), which could have contributed to the convergence of OS curves. These findings should be interpreted cautiously given the exploratory nature of these analyses.

### Clinical implications and future directions

The reasons for non-achievement of J-GCT may help identify older patients who could benefit from more individualized treatment planning. In our cohort, patients aged ≥ 75 years with impaired functional status, ambulation aid use, anticipated omission of lymph node assessment or standard surgery, and intermediate- or high-risk disease in whom adjuvant chemotherapy was being considered or omitted may represent clinically relevant target populations for intervention. In particular, cases attributed to patient preference, advanced age, or concern about postoperative ADL decline may represent subgroups in which additional supportive strategies could be considered. To support more appropriate delivery of guideline-recommended care in older patients, treatment intensity should not be determined by chronological age alone, but rather guided by structured frailty- and function-based evaluation. Implementing a geriatric assessment–guided, multidisciplinary approach could help identify potentially modifiable vulnerabilities before treatment. Targeted prehabilitation, such as nutritional optimization and physical therapy, and enhanced supportive care may improve tolerability for guideline-recommended surgical staging and risk-adapted adjuvant therapy in selected patients. Such proactive strategies may help reduce age-associated gaps in guideline-concordant treatment while aligning its intensity with patients’ goals and overall vulnerability.

In addition, molecular classification may further refine adjuvant treatment selection in older patients with endometrial cancer. The PORTEC-4a trial [23] supported the safety and effectiveness of molecular profile–based individualized adjuvant treatment in patients with high-intermediate-risk endometrial cancer, allowing treatment de-escalation in patients with favorable molecular profiles while identifying those who may require treatment intensification. Incorporating molecular classification may therefore help optimize future guideline-concordant treatment in older patients by balancing recurrence risk, treatment burden, and functional vulnerability.

### Limitations

Several limitations should be noted. First, this was a retrospective, single-institution study, which may limit generalizability. Second, treatment decisions were nonrandomized and individualized, raising the possibility of treatment-selection bias and residual confounding. Although we captured functional status proxies such as ECOG performance status and ADL assistance and adjusted for ECOG performance status in multivariable models, these measures may not fully reflect geriatric vulnerability. In addition, detailed information on frailty, cognition, social support, and reasons for omission of guideline-concordant treatment components was incomplete or unavailable, making it difficult to distinguish undertreatment from appropriate individualized decision-making. Third, subgroup and component-level survival analyses were exploratory and limited by the small number of patients and events. Finally, practice patterns and supportive care may have changed during the study period. Prospective studies incorporating standardized geriatric assessment are needed to validate and extend these findings.

## Conclusions

In this cohort of older patients with endometrial cancer, J-GCT achievement was lower among patients aged ≥ 75 years, with notable gaps in lymph node assessment and risk-adapted adjuvant chemotherapy for intermediate- or high-risk disease. Failure to achieve J-GCT was associated with inferior PFS after adjustment for selected clinical and tumor factors, although this group included heterogeneous treatment patterns. These findings support structured, patient-centered evaluation beyond chronological age in older patients, ideally incorporating geriatric assessment, while balancing vulnerability, competing risks, treatment burden, and goals of care.

## Supplementary information

Below is the link to the electronic supplementary material.

Supplementary file 1 (PPTX 575 KB)

Supplementary file 2 (DOCX 36 KB)

Supplementary file 3 (DOCX 14 KB)

## Data Availability

The datasets generated and/or analyzed during the study are available from the corresponding author upon reasonable request.
