# Supplementary material for: Guideline-concordant treatment and clinical outcomes in older patients with endometrial cancer in Japan: a retrospective single-institution study
Source: Int J Clin Oncol. 2026 Jul 23;31(9):2006–15. doi: 10.1007/s10147-026-03113-z (PMC13506576; doi:10.1007/s10147-026-03113-z)
Supplement: Supplementary file 1 [file 10147_2026_3113_MOESM2_ESM.docx]

Supplementary Table 1. J-GCT achievement by recurrence risk.

| Recurrence risk category |  | Age ≥75  N (%) | Age 70-74  N (%) |
| --- | --- | --- | --- |
| Low risk |  | 17 | 27 |
|  | Achieved J-GCT | 9 (52.9) | 22 (81.5) |
|  | Failed to achieve J-GCT | 8 (47.1) | 5 (18.5) |
| Intermediate risk |  | 14 | 16 |
|  | Achieved J-GCT | 2 (14.3) | 9 (56.3) |
|  | Failed to achieve J-GCT | 12 (85.7) | 7 (43.8) |
| High risk |  | 17 | 28 |
|  | Achieved J-GCT | 5 (29.4) | 21 (75.0) |
|  | Failed to achieve J-GCT | 12 (70.6) | 7 (25.0) |

This table includes only patients who underwent surgery, as recurrence risk was assigned postoperatively.

Supplementary Table 2. Treatment patterns and reasons for non-completion of standard surgery among patients who failed to achieve J-GCT.

|  |  | Total  N (%) | Age ≥75  N (%) | Age 70-74  N (%) |
| --- | --- | --- | --- | --- |
| Patients who failed to achieve J-GCT ^a^ | | 62 | 41 | 21 |
|  | Non-surgery | 11 (17.7) | 9 (22.0) | 2 (9.5) |
|  | Standard surgery; indicated chemotherapy omitted | 6 (9.7) | 2 (4.9) | 4 (19.0) |
|  | Non-standard surgery; chemotherapy administered | 13 (21.0) | 5 (12.2) | 8 (38.1) |
|  | Non-standard surgery; indicated chemotherapy omitted | 19 (30.6) | 17 (41.5) | 2 (9.5) |
|  | Non-standard surgery; no chemotherapy indication | 13 (21.0) | 8 (19.5) | 5 (23.8) |
| Reasons for non-completion of standard surgery ^b^ | | 56 | 39 | 17 |
|  | Due to patient preference | 4 (7.1) | 3 (7.7) | 1 (5.9) |
|  | Due to advanced age | 11 (19.6) | 8 (20.5) | 3 (17.6) |
|  | Due to comorbidities | 3 (5.4) | 2 (5.1) | 1 (5.9) |
|  | Due to advanced stage | 3 (5.4) | 1 (2.6) | 2 (11.8) |
|  | Concern about postoperative ADL decline | 1 (1.8) | 1 (2.6) | 0 (0) |
|  | Unknown | 34 (60.7) | 24 (61.5) | 10 (58.8) |

^a^ Percentages for treatment patterns were calculated among patients who failed to achieve J-GCT.

^b^ Percentages for reasons for non-completion of standard surgery were calculated among patients who did not complete standard surgery.

Indicated chemotherapy refers to adjuvant chemotherapy recommended for intermediate- or high-risk disease according to Japanese guidelines.

Supplementary Table 3. Sensitivity analysis of factors associated with progression-free survival excluding stage from the multivariable model.

|  | Multivariate analysis  (clinical factors + J-GCT) | |
| --- | --- | --- |
| Variables | aHR (95% CI) | *P*-value |
| Age ≥75 years | 1.22 (0.54-2.74) | 0.635 |
| ECOG PS (≥1 vs 0) | 1.21 (0.50-2.90) | 0.672 |
| High-risk histology ^a^ | 4.22 (2.06-8.65) | <0.001 |
| Failure to achieve J-GCT | 2.67 (1.18-6.05) | 0.018 |

aHR, adjusted hazard ratio; CI, confidence interval; ECOG PS, Eastern Cooperative Oncology Group performance status; J-GCT, Japanese guideline-concordant treatment.

Covariate data were missing for high-risk histology (n=5); patients with missing covariates were excluded from univariate and multivariable Cox models.

^a^ High-risk histology was defined as serous, clear cell, or mixed carcinoma with any serous or clear cell component.

Supplementary Table 4. Risk factors associated with progression-free survival excluding patients managed with best supportive care.

|  | Univariate analysis | | Multivariate analysis  (clinical factors) | | Multivariate analysis  (clinical factors + J-GCT) | |
| --- | --- | --- | --- | --- | --- | --- |
| Variables | HR (95% CI) | *P*-value | aHR (95% CI) | *P*-value | aHR (95% CI) | *P*-value |
| Age ≥75 years | 2.21 (1.13 – 4.34) | 0.020 | 2.69 (1.21 – 5.99) | 0.016 | 1.87 (0.78 – 4.47) | 0.159 |
| ECOG PS (≥1 vs 0) | 2.12 (1.04 – 4.33) | 0.039 | 1.11 (0.46 – 2.65) | 0.818 | 0.94 (0.38 – 2.35) | 0.903 |
| Advanced stage (III/IV) | 5.38 (2.75 – 10.50) | <0.001 | 4.33 (1.91 – 9.77) | <0.001 | 4.68 (2.00 – 10.93) | <0.001 |
| High-risk histology ^a^ | 4.17 (2.04 – 8.53) | <0.001 | 2.90 (1.34 – 6.26) | 0.007 | 2.64 (1.18 – 5.91) | 0.018 |
| Failure to achieve J-GCT | 3.36 (1.64 – 7.14) | <0.001 |  |  | 2.83 (1.24 – 6.44) | 0.013 |

aHR, adjusted hazard ratio; CI, confidence interval.

Covariate data were missing for high-risk histology (n=5); patients with missing covariates were excluded from univariate and multivariable Cox models.

^a^ High-risk histology was defined as serous, clear cell, or mixed carcinoma with any serous or clear cell component.

Supplementary Table 5. Risk factors associated with progression-free survival in patients with intermediate- or high-risk disease.

|  | Univariate analysis | | Multivariate analysis | |
| --- | --- | --- | --- | --- |
| Variables | HR (95% CI) | *P*-value | aHR (95% CI) | *P*-value |
| Age ≥75 years | 1.40 (0.63–3.14) | 0.412 | 1.38 (0.47–4.09) | 0.562 |
| Advanced stage (III/IV) | 3.52 (1.58–7.86) | 0.002 | 4.24 (1.76–10.23) | 0.001 |
| High-risk histology ^a^ | 3.57 (1.48–8.62) | 0.005 | 2.86 (1.15–7.09) | 0.023 |
| No adjuvant chemotherapy | 1.75 (0.77–3.96) | 0.178 | 2.37 (0.78–7.14) | 0.126 |

aHR, adjusted hazard ratio; CI, confidence interval.

Covariate data were missing for high-risk histology (n=1); patients with missing covariates were excluded from univariate and multivariable Cox models.

^a^ High-risk histology was defined as serous, clear cell, or mixed carcinoma with any serous or clear cell component.

Supplementary Table 6. Previous studies of treatment patterns and outcomes in older patients with endometrial cancer.

| Study | Country/data source | Study population | N | Study period | Definition of older patients | Main adherence or treatment measure | Key findings |
| --- | --- | --- | --- | --- | --- | --- | --- |
| Felix et al. 2020 (3) | United States; WHI/LILAC survivorship cohort | Postmenopausal women from the WHI cohort who developed endometrial cancer | 629 | EC diagnosed ≥2000; median follow‑up 7.0 years | Age ≥ 75 years | NCCN guideline-concordant treatment | Overall GCT: 83.6%; ≥75 years: 78.5%. GCT was associated with better OS in unadjusted analysis; borderline after adjustment. |
| Ahmed et al. 2008 (4) | United States; SEER-13 registries | Women aged 50 - 95 years with histologically confirmed endometrial cancer | 27,517 | 1992 -2002 | Age ≥ 65:  subgroups 65-74,75-84, 85-95 years | Cancer-directed surgery | Surgery rate: 94% overall; 92.4% in patients aged ≥65 years. Surgery was associated with improved cancer-specific survival. |
| Wright et al. 2011 (5) | United States; SEER-17 registries | Women aged ≥65 years with invasive endometrioid adenocarcinoma of the uterus | 37,718 | 1988 - 2006 | Age ≥ 65 years | Hysterectomy, lymphadenectomy, and radiotherapy | Rates of hysterectomy, lymphadenectomy, and radiotherapy declined with age; older age was associated with worse cancer-specific survival. |
| Yunokawa et al. 2019 (7) | Japan; National Cancer Center Hospital, Tokyo | Patients with endometrial cancer who underwent primary surgery | 531 | 2005-2013 | Age ≥ 65 years:  groups 65–69, 70–74, ≥75 | Standard treatment according to Japanese practice | Standard treatment rates were similar in patients aged 65–74 years but markedly lower in those aged ≥75 years (26%). |
| Tanaka et al. 2022 (13) | Japan; 19 institutions in the Kinki district | Women aged ≥70 years who underwent hysterectomy for endometrial cancer | 1,246 | 2005-2020; follow‑up median 38 months | Age ≥ 70 years | MIS, lymph node resection, and adjuvant therapy | Treatment components, including lymph node resection and adjuvant therapy, were associated with outcomes in selected risk groups. |
| Current study | Japan; single institution | Patients aged ≥70 years with histologically confirmed endometrial cancer | 130 | 2010-2019 | Age ≥ 70 years; comparison of 70–74 vs ≥ 75 years | J-GCT including surgery, standard surgery/staging, and risk-adapted adjuvant chemotherapy | J-GCT achievement was lower in patients aged ≥75 years, and failure to achieve J-GCT was associated with inferior PFS in adjusted analyses. |

EC, endometrial cancer; GCT, guideline-concordant treatment; J-GCT, Japanese guideline-concordant treatment; LILAC, Life and Longevity After Cancer; LN, lymph node; MIS, minimally invasive surgery; NCCN, National Comprehensive Cancer Network; OS, overall survival; PFS, progression-free survival; SEER, Surveillance, Epidemiology, and End Results; WHI, Women’s Health Initiative.
