# Supplementary material for: Guideline-concordant treatment and clinical outcomes in older patients with endometrial cancer in Japan: a retrospective single-institution study
Source: Int J Clin Oncol. 2026 Jul 23;31(9):2006–15. doi: 10.1007/s10147-026-03113-z (PMC13506576; doi:10.1007/s10147-026-03113-z)
Supplement: Supplementary file 2 [file 10147_2026_3113_MOESM3_ESM.docx]

Fig. S1. Treatment cascade by age group.

Flow diagram showing initial treatment (surgery vs non-surgery), standard vs non-standard surgery, recurrence risk category, and adjuvant chemotherapy use. Numbers of patients achieving J-GCT are shown.

Fig. S2. Kaplan–Meier survival curves by recurrence risk category stratified by age group among patients who underwent primary surgery.

(A) Patients aged ≥75 years. (B) Patients aged 70–74 years. Left: Progression-free survival; Right: Overall survival. P values were calculated using the log-rank test.

Fig. S3. Kaplan–Meier survival curves by primary surgery versus non-surgical initial treatment.

(A) Progression-free survival. (B) Overall survival. P values were calculated using the log-rank test. Patients managed with best supportive care were included in the non-surgical group.

Fig. S4. Kaplan–Meier survival curves by J-GCT status among intermediate- or high-risk patients stratified by age group after primary surgery.

(A) Patients aged ≥75 years. (B) Patients aged 70–74 years. Left: Progression-free survival; Right: Overall survival. P values were calculated using the log-rank test.

Fig. S5. Kaplan–Meier survival curves by adjuvant chemotherapy among intermediate- or high-risk patients after primary surgery.

(A) Progression-free survival. (B) Overall survival. P values were calculated using the log-rank test.
